# Je pense donc je fais: transcranial direct current stimulation modulates brain oscillations associated with motor imagery and movement observation

**DOI:** 10.3389/fnhum.2013.00256

**Published:** 2013-06-06

**Authors:** Olivia M. Lapenta, Ludovico Minati, Felipe Fregni, Paulo S. Boggio

**Affiliations:** ^1^Social and Cognitive Neuroscience Laboratory, Center for Healthy and Biological Sciences, Mackenzie Presbyterian UniversitySao Paulo, Brazil; ^2^U.O. Direzione Scientifica, Fondazione IRCCS Istituto Neurologico “Carlo Besta”Milano, Italy; ^3^Laboratory of Neuromodulation, Harvard Medical School, Spaulding Rehabilitation Hospital, Massachusetts General HospitalBoston, MA, USA

**Keywords:** tDCS, EEG, mu rhythm, motor imagery, action observation, primary motor cortex

## Abstract

Motor system neural networks are activated during movement imagery, observation and execution, with a neural signature characterized by suppression of the Mu rhythm. In order to investigate the origin of this neurophysiological marker, we tested whether transcranial direct current stimulation (tDCS) modifies Mu rhythm oscillations during tasks involving observation and imagery of biological and non-biological movements. We applied tDCS (anodal, cathodal, and sham) in 21 male participants (mean age 23.8 ± 3.06), over the left M1 with a current of 2 mA for 20 min. Following this, we recorded the EEG at C3, C4, and Cz and surrounding C3 and C4 electrodes. Analyses of C3 and C4 showed significant effects for biological vs. non-biological movement (*p* = 0.005), and differential hemisphere effects according to the type of stimulation (*p* = 0.04) and type of movement (*p* = 0.02). Analyses of surrounding electrodes revealed significant interaction effects considering type of stimulation and imagery or observation of biological or non-biological movement (*p* = 0.03). The main findings of this study were (1) Mu desynchronization during biological movement of the hand region in the contralateral hemisphere after sham tDCS; (2) polarity-dependent modulation effects of tDCS on the Mu rhythm, i.e., anodal tDCS led to Mu synchronization while cathodal tDCS led to Mu desynchronization during movement observation and imagery (3) specific focal and opposite inter-hemispheric effects, i.e., contrary effects for the surrounding electrodes during imagery condition and also for inter-hemispheric electrodes (C3 vs. C4). These findings provide insights into the cortical oscillations during movement observation and imagery. Furthermore, it shows that tDCS can be highly focal when guided by a behavioral task.

## Introduction

I think, therefore I am. This classic Cartesian statement (“Cogito ergo sum”) could be revised to “I think, therefore I do” to underscore the notion that the motor neural network is as engaged during motor imagery (Jeannerod, 2001; Decety and Grezes, 2006) as during action execution or observation. The mirror neuron system (MNS) plays a significant role in imitation-based learning and action comprehension (Rizzolatti et al., 2001; Rizzolatti, 2005) being part of a system capable of modulating the plan for action execution through mental simulation via observation and internalization of others' actions (Gallese and Goldman, 1998).

It is well established that movement observation and imagery activates supplementary motor area, premotor cortex and primary motor cortex (M1) (Jeannerod, 2001). Additionally, several studies using transcranial magnetic stimulation (TMS) show imagery induced neuroplasticity as indexed by increased motor evoked potentials (MEPs) and reduced motor threshold (MT) (Fadiga et al., 1999; Roosink and Zijdewind, 2010) with muscle-specific activation pattern (Facchini et al., 2002; Fourkas et al., 2006) predominant at the contralateral hemisphere (Fadiga et al., 1999) and long-lasting effects (Pascual-Leone et al., 1995).

Motor activation during action imagery and observation can also be measured through synchronization (ERS) and desynchronization (ERD) of the Mu rhythm (Pfurtscheller and Aranibar, 1977). Initially described by Gastaut and Bert (1954) and commonly detected in the frequency range 8–13 Hz over the sensory-motor cortex (Pineda, 2008), the Mu rhythm is desynchronized during movement execution, observation and imagery. Increases in M1 excitability and consequently Mu desynchronization seem associated with premotor MNS inputs (Jarvelainen et al., 2001; Muthukumaraswamy and Johnson, 2004). This specific neurophysiological signature is observed with action observation (Buccino et al., 2001; Muthukumaraswamy and Johnson, 2004), imagery of self and other's movements (Hari, 2006; Francuz and Zapala, 2011) and in response to static images that induce sensation of movement (Giromini et al., 2010; Pineda et al., 2011).

In order to better understand the role of the motor cortex in imagery, the use of tools to modify cortical excitability (CE)—such as non-invasive brain stimulation—is desirable. For instance, anodal transcranial direct current stimulation (tDCS) of the motor cortex enhances CE while cathodal tDCS decreases it (Nitsche and Paulus, 2000, 2001). Furthermore, previous studies have shown that cathodal tDCS coupled with motor imagery leads to decreased MEP while anodal tDCS induces the opposite results (Quartarone et al., 2004). Conversely, combining anodal tDCS with motor observation leads to long-lasting attenuation of neuropathic pain (Soler et al., 2010); therefore the study of combined approach may also provide initial data for a development of novel therapeutic tools.

Here, we aimed to investigate neurophysiological changes as indexed by Mu rhythm associated with combination of tDCS over M1 with movement observation and imagery tasks. Considering the neuromodulatory effects of tDCS we hypothesized that anodal tDCS might increase Mu ERD and cathodal tDCS would decrease it while sham condition should result in the typical Mu rhythm changes associated with motor observation and imagery. Finally, we hypothesized that tDCS induced Mu rhythm would be similar for both movement observation and imagery.

## Materials and methods

### Participants

Twenty-one right-handed (as verified by the Edinburgh laterality inventory) males (mean age 23.8 ± 3.1) participated in the study which was approved by the institutional ethics committee of the Mackenzie Presbyterian University, Brazil and by the National Ethics Committee (SISNEP, Brazil; CAAE no 0117.0.272.000-11). All participants gave written informed consent.

### Procedure

All participants received on different days with an interval between sessions of at least 48 hours, sham, anodal and cathodal tDCS. Current was ramped-up for 20 s until it reached 2 mA; stimulation was then given for 20 min, and finally the device was turned-off with a ramp-down of 20 s. Electrodes were 35 cm^2^, therefore, the current density was 0.057 mA/cm2. Active electrode was positioned over the left M1 (C3 according to the EEG 10–20 system) and reference electrode at the supraorbital area (SO) as shown to be the optimal area for M1 stimulation according to neurophysiological, behavioral and modeling studies (Nitsche and Paulus, 2001; Fregni et al., 2005; Foerster et al., 2012). tDCS sessions were randomized and counterbalanced between participants.

For sham tDCS, current was ramped-up for 20 s until it reached 2 mA, then ramped-down in 20 s and turned off without participant knowledge so that the participants felt the same sensation of active stimulation. This procedure has been extensively used and shown to be effective in sham-controlled studies (Nitsche et al., 2008).

**Figure 1:**
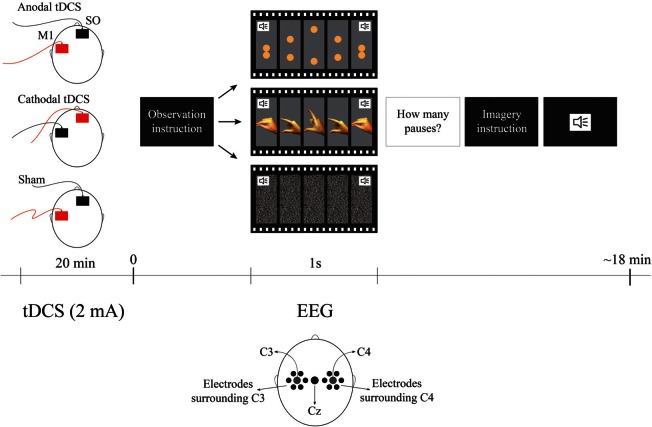
**Experimental Design.** The order of observation videos was random and always followed by the imagery correspondent condition. The surrounding electrodes are: surround C3 (EGI electrodes: 29, 30, 35, 37, 41, 42), Cz, surround C4 (EGI electrodes: 87, 93, 103, 105, 110, 111). The sound symbols represent the moment when the metronome stimulus occurs pacing each cycle. For tDCS conditions red represents the anode and black represents cathode. SO represents the supraorbital (reference) area.

Immediately after tDCS, we positioned the EEG net on the participant (this step took approximately 10 min). The participant sat in a comfortable chair at a 110 cm distance from the computer monitor and received detailed instructions before task performance (the experimental design is illustrated in Figure 1).

### Observation and imagery task

The task was comprised of two experimental conditions—observation and imagery—each one having two types of movement—biological and non-biological. The biological observation blocks were videos lasting 80 s, each showing 1 Hz cycles of opening and closing pincer movements of a male right hand. The maximum aperture between the index finger and the thumb was 7.5 cm. For the biological imagery blocks, they were asked to mentally simulate their own right hand in the same movement, pacing at the same rate as it was shown in the observation condition. Similarly, the non-biological observation blocks were videos of 80 s each showing 1 Hz cycles of two spheres moving vertically toward each other and then touching simulating the biological movement. In order to make the non-biological condition closer to the biological one, the maximum distance between the spheres was also 7.5 cm. The diameter of the two spheres was 1.5 cm similarly to the thickness of the distal phalanx as shown in the screen. Finally, the color of the spheres was composed of proportions of Cyan 0%/Magenta 30%/Yellow 70%/Black 0% resulting in a color similar to the presented fingers. For the non-biological imagery blocks, they were asked to mentally simulate the movement of the two spheres paced at the same rate as it was shown in the observation condition. In order to maintain the participant's attention to the task we presented 2–5 pauses of the movement for 1 s in both observation conditions. Participants were asked to mentally count the pauses and report their answers after completing each trial. Both observation and imagery conditions had their own control conditions i.e., for observation control, there was an 80 s video of white noise and for imagery control, participants were asked to imagine the white noise for 80 s. All cycles of 1 Hz for all blocks were accompanied by the sound of a metronome to guide the imagery condition. Each type of block was presented twice in random orders. The observation condition was always presented before their imagery equivalent conditions in order for the participant learn the movement. In summary there was one active condition and two controls for each task (observation and motor imagery)—i.e., (1) the biological motor movement (observation or motor imagery); (2) control 1: non-biological movement and; (3) control 2: no movement control condition. The overall task duration was approximately 18 min.

### EEG recording and data reduction

To investigate the effects of tDCS, we recorded the electroencephalogram using a high-density 128 geodesic sensor net (Electrical Geodesic). We processed the original data as follows: (1) Highpass filtering at 1 Hz and Lowpass filtering at 30 Hz; (2) removal of the first and final 10 s of each trial and segmentation of the remaining 120 s (combination of the 60 s from the two equal trials) in 2 s epochs (as in Oberman et al., 2005), (3) artifact detection (difference >140 μ V between channels above and below the eyes, a difference >55 μ V between channels near the outer canthi, or one or more channels exceeding an amplitude of 200 μ V), (4) re-referencing of scalp potentials to the average reference, (5) baseline correction from 200 ms before each segment. Epochs containing artifacts due to eye blinks, ocular and head movements were automatically rejected. In order to extract the Mu rhythm, we performed a wavelet analyses for the frequency between 8–11 Hz (Francuz and Zapala, 2011) for C3 and C4 and Cz and the electrodes surrounding C3 and C4. Mu ERD was thereafter calculated as a ratio of the power during experimental conditions relative to the respective control condition. This ratio was used to control variability in absolute Mu power as a result of individual differences. Since ratio data are inherently non-normal as a result of lower bounding, a log transform was used for the analysis. This method has been largely used in Mu rhythm analyses (Oberman et al., 2005; Giromini et al., 2010; Pineda et al., 2011).

### Data analyses

Repeated-measures ANOVA were run focusing on specific effects on C3 and C4 or in Cz and the electrodes surrounding C3 and C4. We performed the analysis of the surrounding electrodes taking into consideration previous findings that show a different pattern between C3 and its surrounding area during motor imagery, videlicet ERD and ERS, respectively (e.g., Neuper et al., 2006). For all analyses the dependent variable was the Mu desynchronization index as described earlier. For the main ANOVA we considered the factors condition (observation vs. imagery), movement (biological vs. non-biological), tDCS (anodal vs. cathodal vs. sham), and hemisphere [C3 (EGI number: 36) vs. C4 (EGI number: 104)] and the respective interaction terms. For the secondary ANOVA we considered the factors condition (observation vs. imagery), movement (biological vs. non-biological), tDCS (anodal vs. cathodal vs. sham), and electrodes [surround C3 (EGI number: 29-30-35-37-41-42) vs. surround C4 (EGI number: 87-93-103-105-110-111) vs. Cz] and the respective interaction terms. For the surrounding electrodes ANOVA, we used the mean values of the previously mentioned electrodes (surround C3, surround C4, and Cz values). When appropriate, *post-hoc* comparisons were carried out using Fisher's LSD. Statistical significance refers to a *p* value < 0.05.

## Results

All participants completed the entire experiment. All participants tolerated the stimulation well and no side effects were reported.

**Figure 2:**
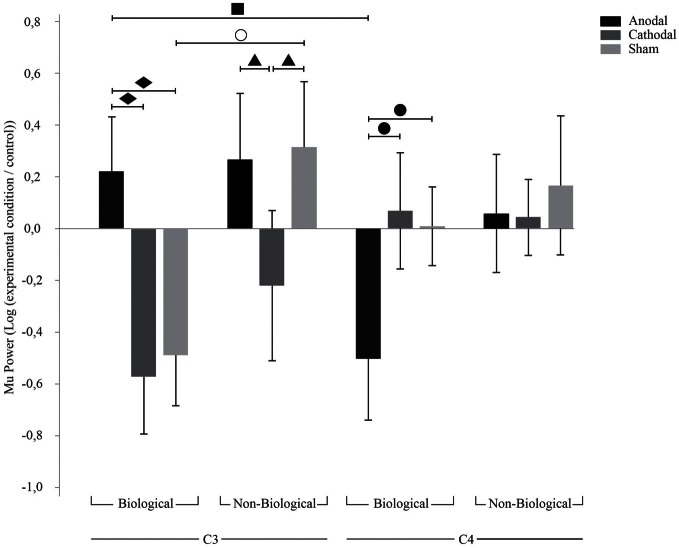
**TDCS effects on C3 and C4.** Bars mean standard errors. ○, sham tDCS resulted in opposing effects for biological and non-biological conditions at C3 (*p* = 0.005); ■, anodal tDCS resulted in ERS at C3 and ERD at C4 (*p* = 0.01); ▲, cathodal tDCS resulted in Mu ERD compared to anodal (*p* = 0.03) and sham (*p* = 0.02) tDCS at C3 during non-biological movement; ●, anodal tDCS resulted in Mu ERD when compared to cathodal (*p* = 0.01) and sham (*p* = 0.02) tDCS at C4 during biological movement; ♦, anodal tDCS resulted in Mu ERS when compared to cathodal (*p* = 0.001) and sham (*p* = 0.002) tDCS at C3 during biological movement.

With regard to the main ANOVA considering C3 and C4 only, we found significant effects for the factor Movement [*F*_(1, 20)_ = 10.2; *p* = 0.005; η^2^_*p*_ = 0.3] and for the interactions tDCS × Hemisphere [*F*_(2, 40)_= 3.4; *p* = 0.04; η^2^_*p*_ = 0.1] and tDCS × Movement × Hemisphere [*F*_(2, 40)_ = 4.2; *p* = 0.02; η^2^_*p*_ = 0.2]. Fisher LSD *post-hoc* analyses considering the factors tDCS and hemisphere revealed significant differences between cathodal and anodal tDCS at C3 (*p* = 0.016). As shown in Figure 2, cathodal tDCS over C3 resulted in significantly larger Mu desynchronization in this area when compared to anodal tDCS independent of the movement type.

*Post-hoc* analyses for the three factors (tDCS, type of movement and hemisphere) revealed significant differences between C3 and C4 during biological movement after sham (*p* = 0.02), anodal tDCS (*p* = 0.001), and cathodal tDCS (*p* = 0.004).

Concerning sham tDCS, we found a typical effect of Mu desynchronization during biological movement in the contralateral hemisphere. There was a significant difference for biological and non-biological movement at C3 during sham tDCS (*p* = 0.005), i.e., Mu desynchronization at C3 during biological movement and Mu increase during non-biological movement. With regard to anodal tDCS, there was an interesting interhemispheric effect: we observed an absence of Mu desynchronization at C3 (hemisphere contralateral to the movement) and simultaneous Mu desynchronization in C4 (hemisphere ipsilateral to the movement) during biological movement only (*p* = 0.01). Cathodal tDCS resulted also in Mu desynchronization at C3 during non-biological movement compared to sham (*p* = 0.02) and anodal tDCS (*p* = 0.03). In addition, during biological movement observation and imagery there was a smaller desynchronization of Mu in the left hemisphere after anodal tDCS compared to sham (*p* = 0.002) and cathodal tDCS (*p* = 0.001), and a significant Mu desynchronization in the right hemisphere (C4) after anodal tDCS compared to sham (*p* = 0.02) and cathodal tDCS (*p* = 0.01).

Our analysis with C3 and C4 electrodes show that effects are not different for movement observation vs. imagery [*F*_(1, 20)_ = 1.05; *p* = 0.32; η^2^_*p*_ = 0.05].

### Analysis of surrounding electrodes

We then analyzed the electrodes Cz and around C3 and C4 (rather than C3 and C4 themselves), ANOVA revealed a significant effect for the interaction tDCS × Condition × Movement [*F*_(2, 40)_ = 3.66; *p* = 0.03; η^2^_*p*_ = 0.15]. Since there was no effect for the hemisphere of the electrodes, the results of this analysis are referred to as surrounding electrodes data.

**Figure 3:**
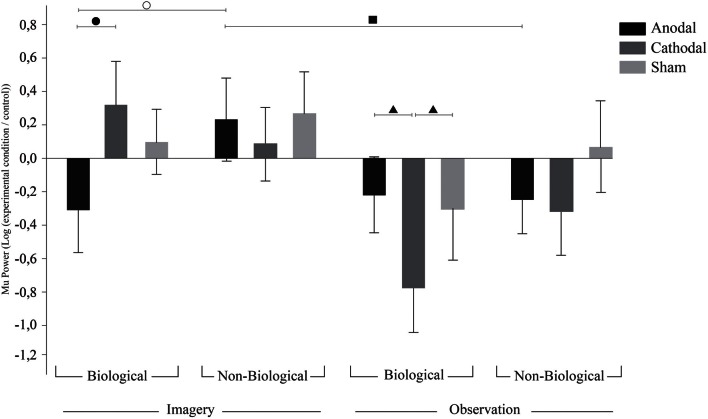
**TDCS effects on surrounding electrodes.** Bars mean standard errors. ○, after anodal tDCS, biological imagery was significantly different from non-biological imagery (*p* = 0.028); ●, anodal tDCS resulted in Mu ERD when compared to cathodal tDCS in biological imagery (*p* = 0.011); ▲, cathodal tDCS resulted in stronger Mu ERD on biological observation when compared to anodal (*p* = 0.023) and sham (*p* = 0.05) tDCS; and ■, anodal tDCS resulted in Mu ERS on non-biological observation when compared to non-biological imagery (*p* = 0.048).

Contrary to the effects found in the C3 and C4 electrodes, Fisher LSD *post-hoc* analysis on surrounding electrodes revealed a tendency for sham tDCS to result in synchronization during biological imagery and desynchronization during biological observation (*p* = 0.09). Also, in the surrounding electrodes, after anodal tDCS we found Mu ERD during biological imagery but not during non-biological imagery (*p* = 0.028). On the other hand, after anodal tDCS we observed Mu ERD during non-biological observation while non-biological imagery provoked Mu ERS (*p* = 0.048). Also, biological imagery had different effects in the surrounding electrodes after anodal and cathodal tDCS resulting in Mu ERD and ERS, respectively (*p* = 0.011). Finally, biological observation led to a contrary effect as cathodal tDCS had a higher magnitude effect on Mu ERD compared to anodal (*p* = 0.023) and sham (*p* = 0.05). There was also a tendency for cathodal tDCS to induce Mu ERD in biological observation but not in non-biological observation (*p* = 0.06), and for Mu ERD and ERS during biological imagery after anodal and sham tDCS, respectively (*p* = 0.09). These results are illustrated in Figure 3.

## Discussion

Motor imagery and observation are extensively correlated with ERD of the Mu rhythm as seen in previous literature. We confirm these findings showing that during biological observation or imagery primed by sham tDCS, the Mu rhythm is desynchronized at the contralateral hemisphere. Here, we extend previous findings showing that active tDCS induced changes in membrane neuronal threshold is associated with modulation of the Mu rhythm during biological movements.

Our sham findings of Mu desynchronization for biological movement despite of condition (observation vs. imagery) corroborate previous neuroimaging and EEG findings (Ruby and Decety, 2001; Muthukumaraswamy and Johnson, 2004; Michelon et al., 2006; Pfurtscheller et al., 2006) and therefore is in line with the mental simulation theory (Jeannerod, 2001; Rizzolatti, 2005). Furthermore, by showing no differences between movement observation and motor imagery, we extended these findings.

With regard to the active tDCS, both cathodal and anodal stimulation interfered with the Mu rhythm elicited by the tasks. However, contrary to our initial hypothesis, we found that anodal tDCS was related to an increase of Mu power (i.e., synchronization) at C3 while cathodal tDCS resulted in a decrease of Mu power (i.e., desynchronization) at C3. Our hypothesis was based on the notion that anodal would facilitate the neurophysiological processes associated with movement observation and imagery (i.e., Mu desynchronization). However, previous non-invasive stimulation research has reported opposite effects for cathodal and anodal tDCS (Antal et al., 2007; Moliadze et al., 2012; Batsikadze et al., 2013). For instance, Batsikadze et al. (2013) applied cathodal tDCS over M1 for 20 min at 1mA and 2mA intensities and showed decreased and increased CE, respectively. Therefore, the intensity and time of stimulation may result in differential effects and our minor ERD effect might be due to a possible cathodal enhancement on CE as recently demonstrated (Batsikadze et al., 2013).

Still, it is not entirely clear which mechanisms underlie these effects and thus caution is necessary in interpreting these data. However, two main explanations seem possible to comprehend our results. On one hand, our findings might be indicative of the notion that Mu ERD is not generated only in the cortical areas. The fact that anodal tDCS induced Mu ERS is in line with the idea that Mu desynchronization is generated by subcortical systems. Leocani et al. (2005) investigated a sample of patients with multiple sclerosis and showed that ERD during programming of voluntary movement are likely mediated by cortical-subcortical connections as they found delayed ERD onset in patients with more severe subcortical damage. This novel finding might be indicating that simple, learned movements (such as opening and closing of the fingers) are mainly generated by subcortical systems and increased cortical activity may interfere with performance of such movement—here we provide such evidence with neurophysiological data. Similarly Antal et al. (2007) reported decrease of CE when combining hand motor contraction with anodal tDCS, which is aligned with our data considering the similar activation during motor observation, imagery, and execution. On the other hand, these effects might also be due to homeostatic mechanisms. The depolarizing effect of anodal tDCS might have established an enhanced CE at C3 in the baseline which induced a paradoxical effect during the task (a homeostatic effect) resulting in Mu ERS. This mechanism might be useful for neural protection as it avoids a high excitability alteration of the area and possible de-stabilization of neural network properties (Abbott and Nelson, 2000). This hypothesis is strengthened by the reverse effect after cathodal stimulation during biological movement, and furthermore, explains our controversial effect of Mu ERD after cathodal tDCS during non-biological movement. This is particularly underscored by the observation of increased ERS during sham tDCS for the non-biological condition compared to control.

Supporting the homeostatic hypothesis, some previous studies have shown reverse effects of tDCS and TMS resulting from: the combination of both techniques (Siebner et al., 2004), non-invasive brain stimulation with drugs, (Fregni et al., 2006; Kuo et al., 2008) and non-invasive brain stimulation with motor task (Antal et al., 2007). Siebner et al. (2004) found that cathodal tDCS followed by 1 Hz TMS results in a facilitatory effect which is in opposition to commonly reported findings in low frequency TMS studies. In turn, Fregni et al. (2006) found that low-frequency 1 Hz rTMS led to CE enhancement in patients with juvenile myoclonic epilepsy presenting with high plasma valproate level (a drug that reduces CE) and CE decrease for the patient group with low valproate level and control group. In addition, Kuo et al. (2008) showed that combining d-cycloserine with anodal tDCS yielded longer reaction times in a sequential motor learning task. Mainly, Antal et al. (2007) reported decreased CE when combining hand motor contraction with anodal tDCS. This is very aligned with our data considering the similar activation during motor observation, imagery, and execution.

As previously mentioned, caution is necessary when interpreting these data as it is known that tDCS can have effects on subcortical (Polania et al., 2012a) and cortical structures (Polania et al., 2012b) through M1 connections. Further studies testing both our hypotheses are necessary to fully comprehend the generating source of the Mu rhythm, and thus, make possible a more accurate interpretation of these neuromodulatory results.

Contrary to our findings, Matsumoto et al. (2010) reported increased CE and Mu ERD after 10 min of 1 mA anodal tDCS and decreased CE and Mu ERS after cathodal tDCS compared to sham. The differences in the results between our study and Matsumoto's may be explained by methodological differences. In particular, the number of participants has major impact in Mu ERD studies since there is a high variability between participants in Mu oscillatory pattern (Pfurtscheller et al., 2006). Matsumoto and colleagues ran the experiment with six participants whereas we had 21 volunteers. Moreover, we selected the electrodes a priori based on previous literature while Matsumoto et al. (2010) made a posterior selection considering electrodes presenting the higher Mu desynchronization. This method rendered results from Matsumoto's article less specific (as they could not analyze the contrast between C3/C4 and surrounding electrodes—that we showed to be important) and the *post-hoc* selection of electrodes increases the type I error in this study. In addition, another study which demonstrated relative contradictory results is the one from Quartarone et al. (2004) which showed no increase in CE after anodal tDCS companied to motor imagery, arguing that one of the strategies is enough to result in ceiling effect. However, by showing that anodal tDCS does not induce motor imagery increased excitability supports our findings to some extent. Moreover, as it is expected that tDCS alone increases CE, the effect of mental imagery blocking this increase in excitability of anodal tDCS supports our results.

Besides the focal effect under the application area of tDCS, we found interesting hemispheric differences. We analyzed Mu characteristics in C3, C4, and Cz and the electrodes surrounding C3 and C4. One important point here is that, as shown by computational model studies, tDCS has a modulatory effect over a large area application and has also a diffuse widespread action due to the distance between electrodes (Datta et al., 2009). Interestingly, despite of the relative non-focal electrical currents induction by tDCS we could observe specific and opposing effects for C3 and surrounding electrodes during biological imagery. Furthermore, this effect was absent during biological observation (except after anodal tDCS). Finally, we also found opposing effects when comparing the contralateral vs. ipsilateral hemisphere (relative to the movement)—i.e., C3 vs. C4; which is in agreement with interhemispheric transcallosal modulation effects as shown behaviorally by other studies (Fregni et al., 2005; Mansur et al., 2005). These results support previous neurophysiological studies showing relatively focal effects of tDCS (Nitsche et al., 2007). Our data extend this finding and demonstrate that tDCS effects can be focalized based on the behavioral task.

The focal ERD/ surrounding ERS theory hypotheses that Mu synchronization occurs to deactivate networks not related to the task (Suffczynski et al., 1999). This hypothesis is supported by our findings as Mu desynchronization over cortical area specifically related to the task followed by an increase of Mu power in motor areas not involved in the task has been previously demonstrated (Pfurtscheller and Neuper, 1994; Neuper et al., 2006; Pfurtscheller et al., 2006).

Interestingly, we found the surrounding ERS effects only during the imagery condition. Similar to our findings, Neuper et al. (2006) have demonstrated surrounding ERS during imagery tasks but not in execution tasks. They registered Mu oscillations over C3 and Cz during cube manipulation and imagery and during foot continuous movement execution and imagery. All tasks showed focal desynchronization, i.e., Mu desynchronization at Cz for foot-related tasks, and at C3 for hand-related tasks. Furthermore, they found Mu synchronization at the area not related to the task during imagery but not execution (e.g., Mu power increase in C3 during imagery of foot movement). Accordingly Pfurtscheller et al. (2006) registered Mu oscillations during 4 types of kinesthetic imagery in the following areas: right hand, left hand, both feet, and thong. They observed Mu ERD in C3 and C4 during both hand motor imagery with evident contralateral dominance and Mu ERD in Cz during feet motor imagery. Also, they report Mu ERS in C3 and C4 (hand area) during motor imagery of feet and tongue. As reported, we found surrounding inhibition for biological imagery but not for biological observation condition. Therefore, it seems plausible that the surrounding inhibition, which reflects an idling or inhibitory state with low cortical neurons excitability (Klimesch et al., 2007), may occur due to removal of motor attention from one modality to another.

The previous studies argued that the execution tasks do not require high directional attention while motor imagery does; thus having a larger focal effect. The high direction of attention seems to be the underlying process of focal Mu ERD associated with Mu surrounding ERS; therefore, our results along with previous literature suggest that motor imagery may have a more focal neuroplastic effect and may be used in rehabilitation approaches. This process seems beneficial to selectively focus and boost the specific recruited area. Considering the aforementioned findings and ours, it is plausible that the imagery tasks demand sustained and focal attention due to their higher complexity as compared to the automaticity of activation during observation. Insofar, observation tasks demand less concentration and can be automated like execution (Neuper et al., 2006).

Our study presents some limitations. Recently, O'Connell et al. (2012) demonstrated that participants receiving 2 mA tDCS judged correctly, more than chance, which stimulation they were receiving (sham vs. active tDCS). We did not directly assess our participants with regard to blinding, therefore possible effects due to correctly guessing which tDCS was being applied might be expected. However, our experiment consisted of three tDCS sessions (two active and one sham) and our results clearly differentiate between the two active conditions—anodal and cathodal effects. Additionally, our methods of ramp up and ramp down of tDCS are similar to previous findings that shows 2 mA as an effective blinding while O'Connell et al. (2012) used 5 s for each ramp up and down. Finally, females composed 75% of O'Connell and colleagues sample; in our experiment, all participants were males. Despite not being directly assessed by previous tDCS experiments, gender effects on tDCS perception may be expected considering previous experiments have shown differential gender effects on pain perception (for a review see Fillingim et al., 2009). A second limitation is that our experiment was performed in a sample of healthy males. Since the effects of tDCS in some tasks appear to differ according to gender (Boggio et al., 2008; Lapenta et al., 2012) further studies should test possible differing effects in females. Furthermore, these results could differ in populations with atypical M1 activation such as psychogenic paresis patients (Liepert et al., 2009, 2011) and autistic patients (Oberman et al., 2005; Théoret et al., 2005; Bernier et al., 2007). Thus, further studies comprising tDCS, EEG, and motor observation and imagery are suggested for better knowledge of possible benefits in populations with atypical brain activation.

In sum, the characteristics of our experiment provide a key differentiation as they allow multiple controls for the effects of the experiment on specific movements and conditions and also on different areas activations in accordance with their involvement in the task. Therefore, our setup allowed evaluation of oscillatory pattern differences relative to the complex task with focal and sustained attention (e.g., imagery) and automated tasks that require less concentration (e.g., observation) extending reports of surrounding effects for movement execution, also considered automatized when compared to imagery. Furthermore, we have shown that tDCS is able to alter Mu rhythm pattern and also that active tDCS priming effects depend on the applied polarity, type of movement, condition, and hemisphere thus introducing a new perspective to the effects of this brain stimulation tool. These novel findings also provide fresh insights regarding possible clinical applications of combined tDCS and motor imagery.

### Conflict of interest statement

The authors declare that the research was conducted in the absence of any commercial or financial relationships that could be construed as a potential conflict of interest.
